# An Osteoprotegerin Gene Polymorphism Is Associated with an Increased Risk of Hip Fracture in Japanese Patients with Rheumatoid Arthritis: Results from the IORRA Observational Cohort Study

**DOI:** 10.1371/journal.pone.0104587

**Published:** 2014-08-08

**Authors:** Shinji Yoshida, Katsunori Ikari, Takefumi Furuya, Yoshiaki Toyama, Atsuo Taniguchi, Hisashi Yamanaka, Shigeki Momohara

**Affiliations:** 1 Institute of Rheumatology, Tokyo Women's Medical University, Tokyo, Japan; 2 Department of Orthopaedic Surgery, Keio University School of Medicine, Tokyo, Japan; IPO, Inst Port Oncology, Portugal

## Abstract

**Introduction:**

Patients with rheumatoid arthritis (RA) have a higher prevalence of osteoporosis and hip fracture than healthy individuals. Multiple genetic loci for osteoporotic fracture were identified in recent genome-wide association studies. The purpose of this study was to identify genetic variants associated with the occurrence of hip fracture in Japanese patients with RA.

**Methods:**

DNA samples from 2,282 Japanese patients with RA were obtained from the DNA collection of the Institute of Rheumatology Rheumatoid Arthritis cohort (IORRA) study. Six single nucleotide polymorphisms (SNPs) that have been reported to be associated with fractures in recent studies were selected and genotyped. Forty hip fractures were identified with a maximum follow-up of 10 years. The genetic risk for hip fracture was examined using a multivariate Cox proportional hazards regression model.

**Results:**

The risk analyses revealed that patients who are homozygous for the major allele of SNP rs6993813, in the *OPG* locus, have a higher risk for hip fracture (hazard ratio [95% CI]  = 2.53 [1.29–4.95], *P*  = 0.0067). No association was found for the other SNPs.

**Conclusions:**

Our results indicate that an *OPG* allele is associated with increased risk for hip fracture in Japanese patients with RA.

## Introduction

Rheumatoid arthritis (RA) is a complex polygenic disorder of unknown etiology characterized by chronic inflammation and joint destruction, resulting in deteriorated physical function and increased risk of falling. [1]–[3] Patients with RA have a higher prevalence of osteoporosis and hip fracture than healthy individuals. [4]–[6] Several clinical risk factors for osteoporotic fracture in RA patients have been reported thus far. [7] Bone mineral density (BMD) is the major predictor of osteoporotic fracture, [5] and the fracture risk assessment tool was developed based on the use of clinical risk factors, including the presence of RA. [8] Recently, multiple genetic risk loci for BMD and osteoporotic fracture were identified in a meta-analysis of genome-wide association studies in people of European descent. [9] However, it is unknown if these genetic risk loci may contribute to fracture risk in non-white populations or in patients with RA. The purpose of the present study was to validate the association and to identify genetic variants associated with the occurrence of hip fracture in Japanese patients with RA. We hope that results from this study will increase our understanding of the genetic basis of hip fracture.

## Materials and Methods

### Study population

Tokyo Women's Medical University Genome Ethics Committee approved the present study (217C) and each individual signed an informed consent form after receiving a verbal explanation of the study; the Genome Ethics Committee also approved consent procedure. This study was a part of the Institute of Rheumatology, Rheumatoid Arthritis cohort (IORRA) study, a single-institution–based, large-scale, prospective observational cohort study with an enrollment of over 5,000 Japanese patients with RA, which began in 2000. [7], [10], [11] DNA samples from 2,282 Japanese patients with RA were obtained from the IORRA DNA collection. All of the patients satisfied the American College of Rheumatology 1987 revised criteria for the classification of RA.

### SNP selection and genotyping

Six single nucleotide polymorphisms (SNPs) reported to be associated with fractures in the recent European study [9] were selected and genotyped: rs6993813, in the osteoprotegerin (*OPG*) locus; rs6696981, in the zinc finger and BTB domain containing 40 (*ZBTB40*) locus; rs3130340, in the major histocompatibility complex (*MHC*) locus; rs3018362, in the receptor activator of the nuclear factor-κB (*RANK*) locus; rs1189505, in the spectrin β nonerythrocytic 1 (*SPTBN1*) locus; and rs2306033, in the low-density lipoprotein receptor-related protein 4 (*LRP4*) locus. Genotyping was performed using the TaqMan fluorogenic 5′ nuclease assay according to the manufacturer's instructions (Applied Biosystems, Tokyo, Japan). [11] Duplicate samples and negative controls were included to ensure accuracy of SNP genotyping. All polymerase chain reactions were performed using the GeneAmp PCR System 9700 (Applied Biosystems), and endpoint fluorescent readings for TaqMan assays were done on an ABI PRISM 7900 HT Sequence Detection System (Applied Biosystems), as described elsewhere. [11]

### Assessment of hip fracture

The occurrence of hip fractures after enrollment in IORRA was determined from the responses to a patient questionnaire every 6 months from October 2000 to October 2010, with a maximum follow-up period of 10 years (median, 7.5 years). The data were confirmed by review of medical records and radiographs, as described elsewhere. [7], [12] Only the occurrence of the first hip fracture reported by patients was included in this study. Hip fractures caused by major trauma such as car accidents were excluded. A total of 40 hip fractures in 40 patients were identified and included in this study.

### Statistical analyses

This study used longitudinal data analyses to define whether the reported risk allele of each SNP was associated with the occurrence of hip fracture in Japanese RA patients. [9] The length of time from the date of enrollment in IORRA to the date of occurrence of hip fracture was calculated. A multivariate Cox proportional hazards model was used to examine the association of risk alleles of each SNP with the occurrence of hip fracture in 2,282 patients. Adjustments were made for the following independent non-genetic risk factors: age, body mass index, the Japanese version of the Health Assessment Questionnaire disability score, and history of total knee replacement, which we have previously shown to be clinical risk factors for the occurrence of hip fracture. [7] The proportional hazards assumption for the Cox model was assessed using log-minus-log plots for survival analysis. All statistical analyses were performed using the R software package (http://www.r-project.org/).

## Results

Demographic, clinical, and therapeutic data of 2,282 patients at the time of enrollment in IORRA are shown in Table 1. The overall genotyping success rate was 97.5% and the genotyping concordance rate was 100% as assessed by duplicate samples. After the application of quality control criteria for genotyping (removal of samples that consistently failed for >16.7% [1/6] SNPs and removal of SNPs with a call rate <95% after removing samples that consistently failed), 2,210 of the 2,282 samples and all SNPs were accepted for the analyses. The studied SNPs were found to be in Hardy-Weinberg equilibrium.

**Table 1:** Demographic, clinical and therapeutic data at the time of an enrollment in IORRA.

Factor	median (interquartile range) or n (%)	
Age, years	57.0	(48.5–64.2)
Sex, female	1949	(85.4)
Duration of disease, years	7.0	(2.0–13.0)
BMI, kg/m^2^	21.1	(19.3–23.2)
DAS28	4.2	(3.4–5.1)
J-HAQ	0.8	(0.3–1.4)
RF, positive*	1801	(82.2)
ACPA, positive†	1908	(86.8)
History of smoking, ever	755	(34.5)
History of TKR, ever	99	(4.3)
DMARDs use, ever	1949	(85.4)
Methotrexate use, ever	980	(43.2)
Biologic use, ever	45	(2.0)
Corticosteroid use, ever	1119	(49.0)
Bisphosphonate use, ever	93	(4.1)
Active vitamin D use, ever	82	(3.6)

*Maximum value of RF measured in the cohort project during 2000–2010 for each individual was used.

† Cut-off  = 4.5 IU/ml.

IORRA, Institute of Rheumatology Rheumatoid Arthritis cohort study; BMI, body mass index; DAS28, disease activity score in 28 joints; J-HAQ, the Japanese version of Health Assessment Questionnaire; RF, rheumatoid factor; ACPA, anti-citrullinated peptide antibody; TKR, total knee replacement; DMARDs, disease modifying antirheumatic drugs.

**Figure 1:**
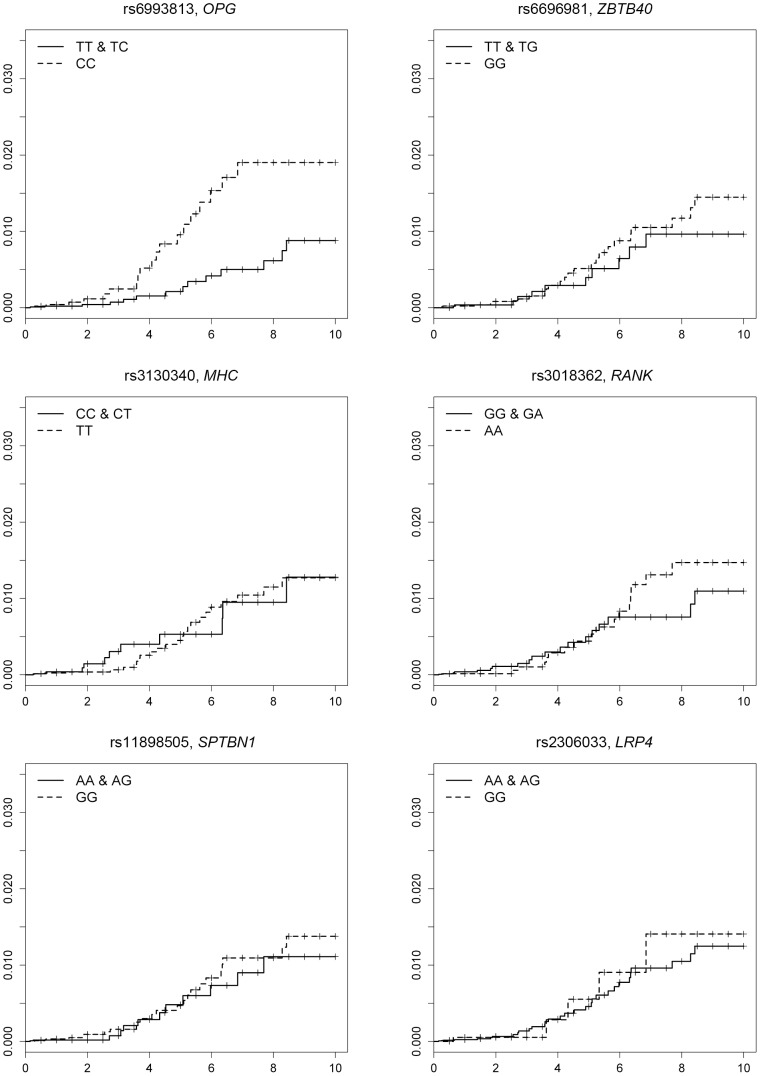
Cumulative incidence of hip fracture for patients who were homo- or heterozygous for the non-risk allele and patients homozygous for the risk allele of each single nucleotide polymorphism (by the Kaplan-Meier method). Homozygous for the risk allele of rs6993813 (C) in the *OPG* locus was significantly associated with the occurrence of hip fracture (*P* = 0.0067).

A multivariate Cox proportional hazards model revealed that patients who are homozygous for the major allele of SNP rs6993813, in the *OPG* locus, had a higher risk for hip fracture compared to other patients (HR [95% CI]  = 2.53 [1.29 to 4.95], *P*  = 0.0067 [α  = 0.0083 after Bonferroni correction], Table 2, Figure 1). No associations were found for the other SNPs (Table 2).

**Table 2:** Multivariate Cox proportional hazards model of each SNP associated with the occurrence of hip fracture.

Locus	SNP	MAF*	Risk allele	HR (95%CI)	*P*
*OPG*	rs6993813	0.423 (C/T)	C	2.53 (1.29–4.95)	0.0067
*ZBTB40*	rs6696981	0.218 (G/T)	G	1.42 (0.70–2.87)	0.33
*MHC*	rs3130340	0.140 (T/C)	T	0.78 (0.38–1.58)	0.48
*RANK*	rs3018362	0.312 (A/G)	A	1.12 (0.59–2.14)	0.73
*SPTBN1*	rs11898505	0.216 (G/A)	G	1.28 (0.64–2.55)	0.48
*LRP4*	rs2306033	0.363 (A/G)	G	1.05 (0.41–2.69)	0.094

All analyses were adjusted for independent non-genetic factors: age, body mass index, Japanese version of Health Assessment Questionnaire disability score, and history of total knee replacement. [7].

*Alleles are listed as major allele/minor allele.

SNP, single nucleotide polymorphism; MAF, minor allele frequency; HR, hazard ratio; CI, confidence interval; *OPG*, osteoprotegerin; *ZBTB40*, zinc finger and BTB domain containing 40; *MHC*, major histocompatibility complex; *RANK*, receptor activator of the nuclear factor-κB; *SPTBN1*, spectrin β nonerythrocytic 1; *LRP4*, low-density lipoprotein receptor-related protein 4.

## Discussion

This study demonstrated that a polymorphism of rs6993813 in *OPG* was significantly associated with the occurrence of hip fracture in Japanese patients with RA. To date, many genome-wide association studies have identified multiple genetic risk loci for osteoporosis and osteoporotic fracture. [9], [13] We recently identified that a *GC* polymorphism related to serum vitamin D concentration was significantly associated with the occurrence of hip fracture. [12] However, the genetics of fracture remain poorly understood in the Asian population, and genetic constitution may differ among populations. [14], [15] Therefore, it is important to validate whether the previous findings are relevant to other ethnic groups.

The *OPG* gene encodes known regulators of bone remodeling and maintains a fine balance between bone formation and resorption involving osteoblasts and osteoclasts, potentially contributing to pathophysiological mechanisms of osteoporosis and osteoporotic fracture. [16] OPG is a member of the RANK-RANKL-OPG signaling pathway and is important to BMD genetics, and appears to have an influence on fracture risk. [17] Indeed, therapeutic targeting of the RANK-RANKL-OPG signaling pathway clearly influences fracture risk. [18]

The strength of this study is that the samples were relatively large and followed up for comparatively long periods. The data were based on a single-institution cohort study of Japanese patients with RA. Therefore, the differences between regions, races, and treatment strategies are expected to have had minimal influence on the results.

The possible limitation of this study is that we could not evaluate the influence of BMD because the data were not available for most patients. A bigger sample size could increase the statistical power to detect minor effects on events. It is likely that we were not able to validate associations of other genes and fracture risk due to the insufficient statistical power of this study. Further studies are required to confirm these associations and dissect the fracture risk, independently of BMD.

In conclusion, our data demonstrated that rs6993813 in *OPG* was significantly associated with increased risk of hip fracture in Japanese patients with RA. These results should contribute to a better understanding of bone metabolism and establishment of models for prediction of fracture risk in RA patients.
